# MiST 4.0: a new release of the microbial signal transduction database, now with a metagenomic component

**DOI:** 10.1093/nar/gkad847

**Published:** 2023-10-04

**Authors:** Vadim M Gumerov, Luke E Ulrich, Igor B Zhulin

**Affiliations:** Department of Microbiology and Translational Data Analytics Institute, The Ohio State University, Columbus, OH 43210, USA; Ulritech, LLC, Mount Pleasant, SC 29466, USA; Department of Microbiology and Translational Data Analytics Institute, The Ohio State University, Columbus, OH 43210, USA

## Abstract

Signal transduction systems in bacteria and archaea link environmental stimuli to specific adaptive cellular responses. They control gene expression, motility, biofilm formation, development and other processes that are vital to survival. The microbial signal transduction (MiST) database is an online resource that stores tens of thousands of genomes and allows users to explore their signal transduction profiles, analyze genomes in bulk using the database application programming interface (API) and make testable hypotheses about the functions of newly identified signaling systems. However, signal transduction in metagenomes remained completely unexplored. To lay the foundation for research in metagenomic signal transduction, we have prepared a new release of the MiST database, MiST 4.0, which features over 10 000 metagenome-assembled genomes (MAGs), a scaled representation of proteins and detailed BioSample information. In addition, several thousands of new genomes have been processed and stored in the database. A new interface has been developed that allows users to seamlessly switch between genomes and MAGs. MiST 4.0 is freely available at https://mistdb.com; metagenomes and MAGs can also be explored using the API available on the same page.

## Graphical Abstract

**Graphical Abstract:**
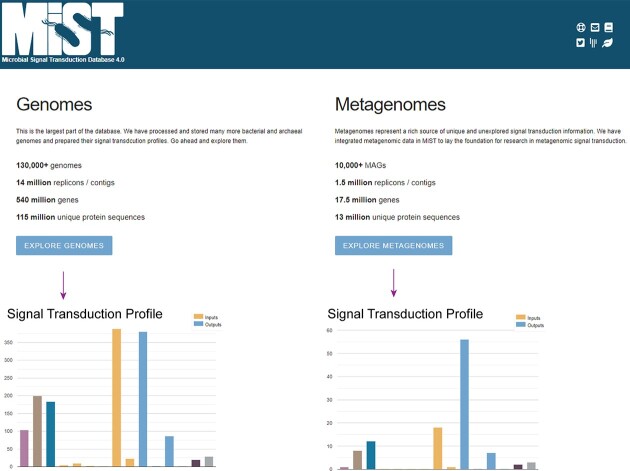

## Introduction

Cellular processes in all living organisms are controlled by signal transduction pathways. Since their discovery (1), two-component regulatory systems were the primary focus of research in bacterial signal transduction (2). In these pathways, the sensory function (signal detection) resides in a histidine kinase, which upon stimulation phosphorylates another protein, a response regulator, which contains a regulatory module (usually a dedicated protein domain) to affect a cellular function. The majority of experimentally characterized two-component systems regulate gene expression (via DNA-binding domains of response regulators), but others control second messenger turnover (via cyclic nucleotide cyclase and phosphodiesterase domains), protein phosphorylation (via serine/threonine kinase and phosphatase domains) and protein–protein interactions. Subsequently, it was recognized that sensory and regulatory domains typical of two-component systems may reside in a single protein. Such proteins were termed one-component systems and showed to be the dominant mode of bacterial signal transduction (3). A highly specialized type of two-component signal transduction, termed chemosensory system, in addition to histidine kinases and response regulators also involves dedicated receptors that contain sensory domains, receptor-modifying enzymes, adaptor/scaffolding proteins and signal-terminating phosphatases (4). The extracytoplasmic function (ECF) sigma factors were also proposed as a special mode of signal transduction (5). The MiST 3.0 database utilized this general classification principle—one-component, two-component (with chemosensory systems as a subcategory) and ECF—that reflects a component design and offers a complete (from sensing to regulation) description of signal transduction systems (6). This principle is used to simplify domain-based rules for automated assignment, not to serve as a golden standard for classification of signal transduction proteins. There is no ‘correct’ way of classifying signal transduction proteins due to their multidomain nature and high connectivity and complexity of signaling pathways. However, the current MiST schema enables the user to have a direct access to the most complete set (as allowed by automated assignment) of signal transduction proteins in a genome(s) of interest for further exploration, including further classification into different functional groups. MiST calculated and stored signal transduction profiles of tens of thousands of reference genomes deposited into the NCBI RefSeq database (7) and served as a helpful unique resource for research on signal transduction in bacteria and archaea. The database has been used in over a hundred projects around the world.

Traditional microbiology and microbial genome sequencing and genomics were based on cultivated clonal cultures. To assess the diversity of a natural sample, early environmental gene sequencing cloned specific genes, in particular the 16S ribosomal RNA gene. These studies revealed that a significant microbial diversity had been hidden from and was unreachable for cultivation-based methods (8). The next achievement is metagenomic sequencing, which revealed an unprecedented microbial diversity of the environmental samples (9,10). Finally, technological advances have made it possible to assemble complete or nearly complete genomes from metagenomic sequences (metagenome-assembled genomes, or MAGs) (11,12). In the last few years, many thousands of MAGs have been reported in the literature, for a variety of environments and host-associated microbiota, including humans (12,13). MAGs have helped us better understand microbial populations and their interactions with the environment where they live. Furthermore, a large portion of MAGs belong to novel species; therefore, they help to decrease the so-called microbial dark matter (14–16).

Until now, computational studies on signal transduction were carried out using genomes of cultivated organisms only and a whole layer of genomic data in the form of MAGs remained unexplored. We have prepared a new release of the database, MiST 4.0, that includes MAGs and allows users to begin exploration of their signal transduction profiles. MAGs represent a rich source of unique and unexplored information, and this release of the database lays the foundation for research in metagenomic signal transduction. Our preliminary analysis revealed both common and unique features in signal transduction trends between cultivated and uncultivated organisms.

## New features and improvements

### Metagenome-assembled genomes

A dedicated MiST module was developed to process and store MAGs. In MiST 4.0, a list of MAGs is obtained from the GOLD database (17) and corresponding GenBank (18) identifiers are supplied to this module. Based on the identifiers, the module next fetches genome assembly information, nucleotide and protein sequences, metadata and taxonomy from the NCBI database and stores the data into the MiST database. Additionally, BioProject and BioSample information (19,20) is obtained from NCBI and stored into the database. For each protein encoded in the genome of a given MAG, the MiST pipeline identifies several protein features: protein domains, low-complexity regions, transmembrane regions, coiled coils and the gene neighborhood of the corresponding gene. This provides a comprehensive picture about protein repertoires and the variety of their types in the processed MAGs. Similar to genomes, signal transduction proteins of MAGs are identified and distributed to corresponding signal transduction categories based on our hierarchical rule system (21) and signal transduction-specific profile hidden Markov models (HMMs) stored in the Pfam database (22) as well as using our own profile HMMs (6). Thus, each MAG has its own comprehensive signal transduction profile. It should be mentioned that the Pfam database now is part of the InterPro database (23).

**Figure 1.**
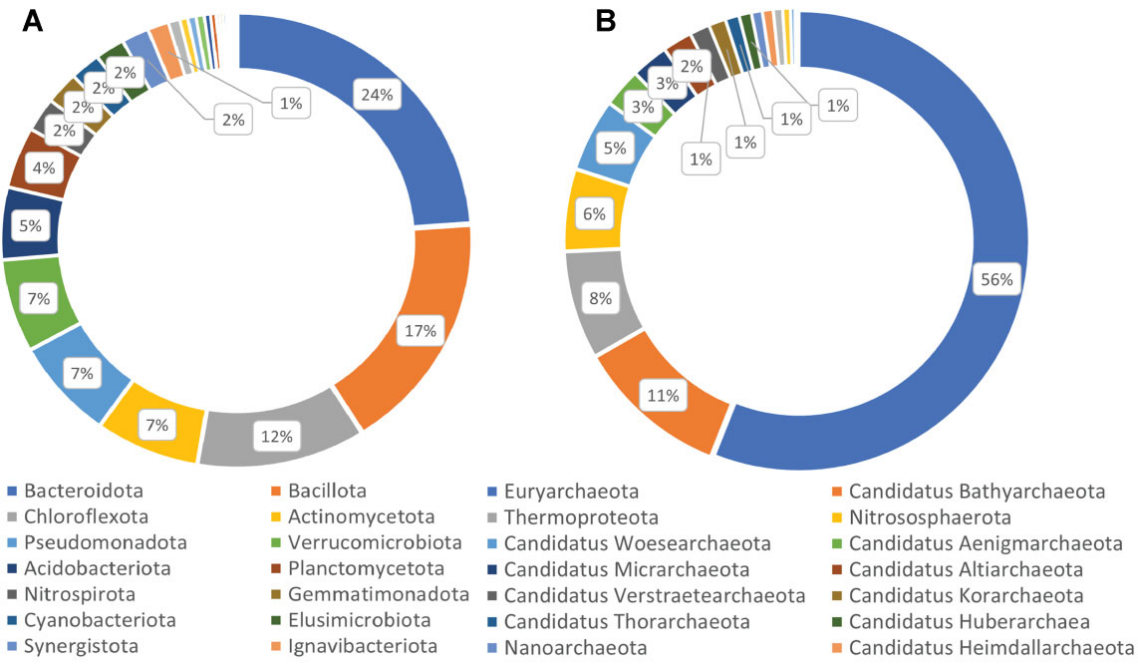
Taxonomic composition of MAGs in MiST 4.0: (**A**) bacteria and (**B**) archaea. Major phyla are listed.

Currently, MiST 4.0 contains 9252 bacterial and 1026 archaeal MAGs from 10 250 biosamples. Comprehensive BioSample information is available for each MAG. The total number of all signal transduction genes across MAGs in MiST is 882 172. MAGs cover 34 bacterial (3909 MAGs) and 4 archaeal phyla (648 MAGs) (Figure 1) and many more uncategorized bacterial and archaeal groups. The majority of them belong to Candidatus and Candidate division phylum-level groups. It is important to emphasize that there are various well-known archaeal groups among them, such as Candidatus Korarchaeota, Candidatus Lokiarchaeota, Candidatus Thorarchaeota, Candidatus Bathyarchaeota and others (Figure 1).

The prokaryotic RefSeq database has three categories of genomes: reference genomes (chosen by the RefSeq curatorial staff based on their quality and importance to the community), representative genomes (for species without a reference genome, a chosen assembly per defined species) and nonrepresentative genomes (the bulk of the RefSeq taxonomically diverse prokaryotic collection). For uploading and processing in the genomic part of MiST, genomes from the RefSeq database are selected based on the priority of the genome category: first, reference and representative genomes are fetched and processed, and then nonrepresentative genomes. On the other hand, in the GOLD database no such categories for MAGs exist; therefore, no special selection criteria are used by the MiST pipeline. MAGs can be explored using either a web interface (https://mistdb.com) or a RESTful API (application programming interface). The MAG database adheres to the same schema as the genomic part of the MiST; therefore, all the API endpoints developed to work with the genomic database can be used in the same fashion to query the Metagenomes database. The Metagenomes API can be accessed at the following URL: https://metagenomes.asc.ohio-state.edu/.

### BioSample information

Genomes and metagenomes have the NCBI BioSample data (19,20) associated with them that include such information as submitter-supplied description, isolation source, geographic location, strain, submitter, anaerobic/aerobic status and other information. Although the BioSample data are standardized, this information varies from species to species and, furthermore, submitters provide the information with varying degrees of completeness and depth. However, these data can be very useful for various types of studies, including comparative analyses. Therefore, in this release of the MiST database, we have included BioSample data for all stored genomes and MAGs. This information can be accessed either from the Genome details page by clicking on the BioSample field in the Genome Summary block or via the API. The data can be used to identify signatures in the signal transduction profiles of organisms isolated from various environments.

### Taxonomy update

Starting from January 2023, NCBI Taxonomy (24) has adjusted prokaryote phylum names in line with the recent inclusion of the rank ‘phylum’ in the International Code of Nomenclature for Prokaryotes (ICSP) using the ending -*ota* for phylum names based on the name of a type genus (25). This involved changes to 42 taxa (25). This also includes the change of several names that have long been in use (such as Firmicutes and Proteobacteria) to newly formalized names (Bacillota and Pseudomonadota, respectively) that may be unfamiliar to some. We have included these changes in MiST 4.0 for both genomes and MAGs. The full list of changes can be found on the MiST help page. In addition, in accordance with the NCBI Taxonomy the phylum Cyanobacteria in MiST 4.0 has been renamed to Cyanobacteriota following the rules of ICSP (26) with a single class Cyanophyceae. We have also made internal changes to two new phyla, Myxococcota and Bdellovibrionota, according to (27) and the NCBI Taxonomy that previously were part of Deltaproteobacteria. Myxococcota includes two classes: Myxococcia with a single order Myxococcales and Polyangia with three orders Haliangiales, Nannocystales and Polyangiales. Bdellovibrionota includes three classes: Bacteriovoracia with a single order Bacteriovoracales, Bdellovibrionia with a single order Bdellovibrionales and Oligoflexia with two orders Oligoflexales and Silvanigrellales. In addition, Epsilonproteobacteria and the former deltaproteobacterial order Desulfurellales now comprise a new phylum, Campylobacterota (27). Within the order Desulfurellales, a new class, Hippeaceae, has been proposed (28) and now included in NCBI Taxonomy, which we also included in the MiST database. Another proposed phylum, Desulfobacterota (27), is currently under the name Thermodesulfobacteriota in NCBI Taxonomy and we keep it under the same name in the MiST database.

### New interface

We have updated the MiST interface to provide seamless integration of genomes and MAGs within a single website. A switcher at the top of the MiST website allows users to easily switch between genomes and metagenomes from any page. The two parts of the database can also be accessed from the home page. Upon selection of the database, the entire navigation infrastructure gets configured for the interaction with the chosen database. This process is seamless and not noticeable by users. Similar to genomes, each metagenome has its own page with a complete signal transduction profile and classification of signal transduction systems.

**Figure 2.**
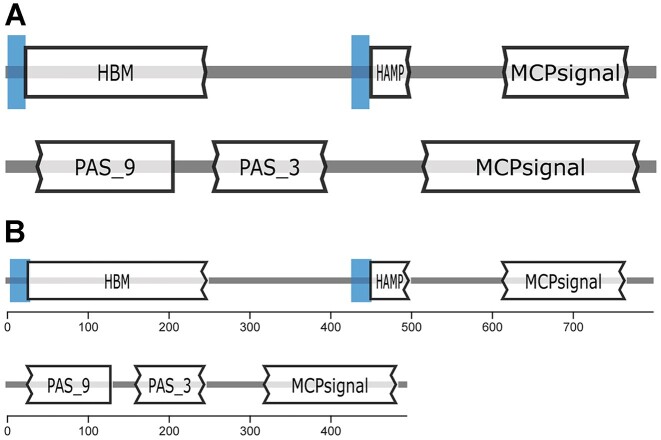
Representation of proteins and their domains in MiST 3.0 (**A**) and MiST 4.0 (**B**). Domains identified using the Pfam database profile HMMs are shown. Blue rectangles denote transmembrane regions.

We have refreshed the color scheme of the website and assigned a different color to the Genomes and Metagenomes parts of the interface to make switching between the databases clear. In this release, we also made the images representing protein sequences scaled according to their lengths and added scale bars below them so that users can easily assess and compare protein sequence lengths (Figure 2). On the protein search results page, the scaling is based on the longest currently displayed protein sequence, while on the protein details page it is based on the predefined maximum length.

To search for an organism, users should enter its name or corresponding genome assembly identifier into the search form. This will initiate the search in several fields in the database, including organism names and all taxonomic levels. The more complete the name is entered, the more accurate the results will be. On the other hand, instead of a specific organism, users may also want to search for all genomes of a given taxonomic level in the MiST database, which can be done by simply entering the taxonomic level. Partial but not abbreviated names will also trigger searches and return results. Once the results are obtained, a genome filter can be used to further narrow the results to the desired level of taxonomy and/or genome assembly.

## Database statistics and preliminary analysis

**Figure 3.**
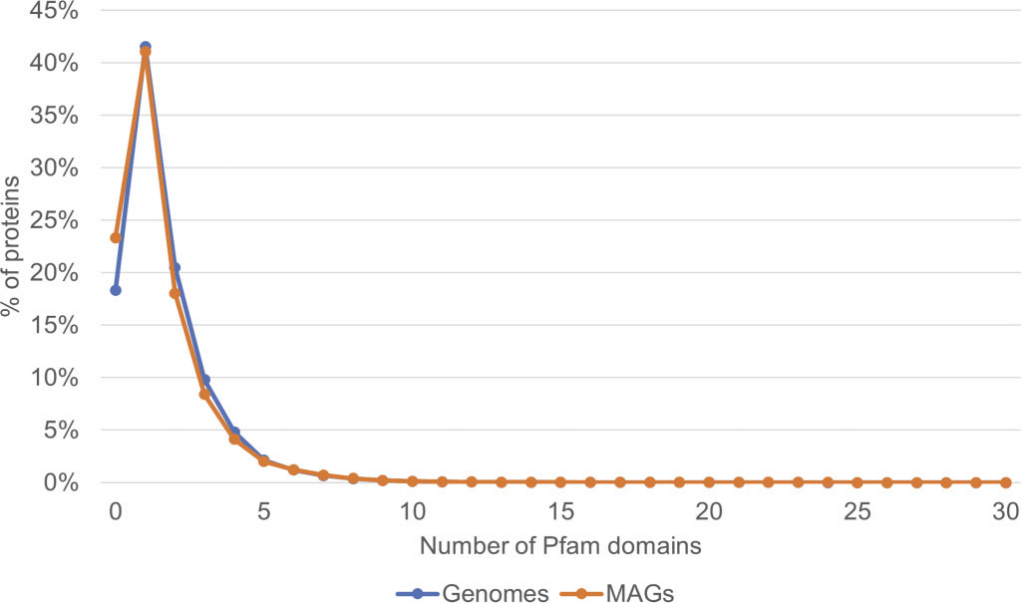
Percentage of proteins versus the number of identified Pfam domains in genomes (blue) and MAGs (orange) in the MiST 4.0 database.

**Figure 4.**
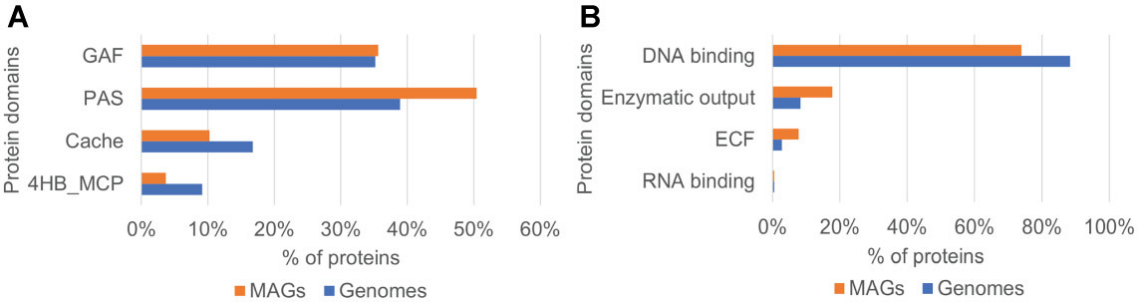
The most abundant input (**A**) and output (**B**) domains in genomes (blue) and MAGs (orange) in the MiST 4.0 database. Domain definitions follow the Pfam database domain nomenclature (InterPro identifiers are in parentheses): GAF (IPR003018), domain found in proteins including cGMP-specific phosphodiesterases, adenylyl cyclases and fhlA; PAS (IPR000014), domain named after Per (period circadian protein), Arnt (Ah receptor nuclear translocator protein) and Sim (single-minded protein); Cache (CL0165), extracellular domain found in a wide range of proteins, including the animal voltage-gated calcium channel subunits and prokaryotic chemotaxis receptors; 4HB_MCP (IPR024478), ubiquitous sensory module four-helix bundle methyl-accepting chemotaxis protein domain. ECF stands for extracytoplasmic function sigma factor domain.

There are currently 131 700 genomes (131 259 biosamples), >540 million genes and >115 million unique protein sequences in the MiST Genomes database. Out of >540 million genes, ∼41 million genes are signal transduction genes. In the MiST Metagenomes database, corresponding numbers are as follows: 10 278 MAGs, >17.5 million genes (including 882 172 signal transduction genes) and >13 million unique protein sequences. We counted the number of Pfam domains [the Pfam domain database (22) is now part of the InterPro database (23)] across unique protein sequences in both Genomes and Metagenomes databases and found similar patterns in domain count distributions (Figure 3). In 99% of proteins, the number of identified Pfam domains is in the range between 0 and 8. Percentages of proteins without any identified Pfam domain are slightly different in genomes versus MAGs: 18% and 23%, respectively. The actual difference can be even larger given the smaller size of the MAG database. This highlights that MAGs contain an additional sequence diversity unreachable if the analysis is confined to genomes. We observed overall similar trends in several aspects of signal transduction in MAGs compared to genomes; however, deviations in certain components were abound as well. Among the most abundant sensor domains and regulatory output domains, proteins with domains from PAS superfamily and DNA binding category, respectively, are the most prevalent in both Genomes and Metagenomes components of the database (Figure 4). However, MAGs on average have more PAS domains than genomes and, conversely, fewer Cache as well as 4HB_MCP domains. The ligand-binding signatures of the Cache and PAS domain superfamilies have recently begun to be deciphered (29–32), which will aid future comparative signal transduction studies. The Cache domain clan of the Pfam database, reclassified in 2016 (33), consists of 23 families of extracellular domains present in a wide range of proteins. Of them, dCache_1 is the largest domain family followed by much smaller families, such as CHASE, sCache_3_2, sCache_2 and others. The Pfam PAS domain clan consists of 17 families of intracellular domains with the largest of them being PAS, PAS_3, PAS_4 and PAS_9. Recently, a refined classification of the clan has been proposed (31). The less studied but widespread 4HB_MCP domain, which has four-helix bundle structure, is currently represented by four families of extracellular domains. MAGs also exhibit a higher proportion of enzymatic output domains and the ECF sigma factors (34) than DNA-binding domains compared to genomes. We would like to stress that in the case of both input and output domains ‘enzymatic’ is a genomic and not a biochemical definition: some of these domains are homologous to enzymes, but do not have enzymatic activity. Currently, the MiST database is not curated and therefore we have no means to make such functional refinements.

**Figure 5.**
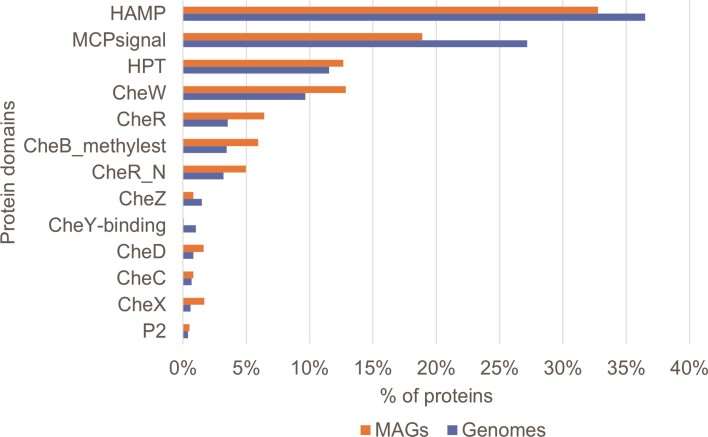
The ratios of various components of chemosensory systems in genomes (blue) and MAGs (orange) in the MiST 4.0 database. Domain definitions follow the Pfam domain nomenclature (InterPro identifiers are in parentheses): HAMP (IPR003660), an ∼50-amino acid α-helical region present in histidine kinases, adenylyl cyclases, methyl-accepting proteins and phosphatases; MCPsignal (IPR004089), methyl-accepting chemotaxis protein (chemoreceptor); HPT (IPR008207), histidine-containing phosphotransfer domain; CheW (IPR002545), domain interacting with the methyl-accepting chemotaxis proteins and relaying signals to CheY response regulator; CheR (IPR022642), an *S*-adenosylmethionine-dependent methyltransferase that methylates the MCP at specific glutamate residues; CheB_methylest (IPR000673), a methylesterase responsible for removing the methyl group from the gamma-glutamyl methyl ester residues in the MCPs; CheR_N (IPR022641), a smaller N-terminal helical domain linked via a single polypeptide connection to a larger C-terminal α/β domain of CheR; CheZ (IPR007439), a phosphatase dephosphorylating CheY; CheY-binding (IPR015162), a domain found in the response regulator histidine kinase CheA, which binds to and phosphorylates a corresponding domain on CheY; CheD (IPR005659), a deamidase that acts on MCPs to augment CheR-mediated methylation; CheC (IPR007597) and CheX (IPR028051), CheY-P phosphatases; P2 (IPR010808), a domain within CheA, to which response regulators bind.

Constituents of the chemosensory system in the two components of MiST have similar ratios (Figure 5). The HAMP domain was found to be the most abundant component, which is not surprising since it is found not only in chemoreceptors but also in two-component systems. The chemosensory-specific overabundant component is MCPsignal domain, which is a signaling domain of chemoreceptors. Chemoreceptors are the most prevalent part of chemosensory machinery: for example, up to 90 chemoreceptors are encoded in the genome of *Caryophanon latum* (35). Interestingly, HAMP and MCPsignal domains are noticeably more prevalent in genomes than in MAGs compared to other chemosensory proteins. This may reflect the fact that MAGs may tend to have fewer chemoreceptors, or fewer chemosensory systems overall, than genomes of cultivated organisms. The HPT domain, which is responsible for transferring a phosphoryl group from CheA to CheY, and the CheW domain necessary for the binding of CheA to chemoreceptors and CheW protein are the next components in terms of prevalence. Methylesterase CheB and methyltransferase CheR are, expectedly, in almost equal ratios in both Genomes and Metagenomes components of the MiST database (see Figure 5). Other components are less abundant. Interestingly, HPT, CheW, CheR, CheB, CheD and CheX are found in higher proportions in MAGs than in genomes.

**Figure 6.**
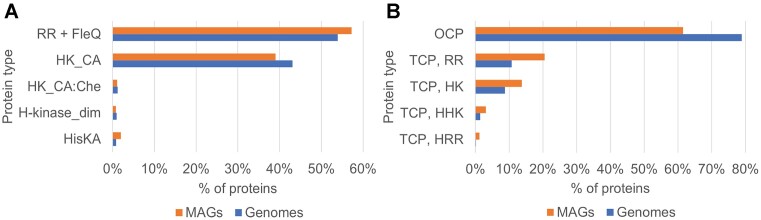
Comparison of key signal transduction components and systems in genomes and MAGs. (**A**) The ratios of histidine kinases and response regulators. (**B**) The ratios of one-component and two-component proteins. Genomes are shown in blue and MAGs in orange. Abbreviations: RR, response regulator; FleQ, response regulator receiver domain; HK_CA, histidine kinase catalytic domain; HK_CA:Che, histidine kinase catalytic domain, CheA-specific; H-kinase_dim, histidine kinase dimerization (P3) domain, CheA-specific; HisKA, histidine kinase dimerization domain; OCP, one-component system protein; TCP, two-component system protein; HK, histidine kinase; HHK, hybrid histidine kinase; HRR, hybrid response regulator.

The prevalence of the catalytic domain of histidine kinases across signal transduction systems is comparable to the prevalence of response regulators in both genomes and MAGs, though it is more pronounced in genomes (Figure 6A). The calculated database statistics also supported the conclusion of the 2005 paper (3) about the prevalence of one-component systems over two-component systems (Figure 6B). This trend is observed in both Genomes and Metagenomes components of MiST, which shows that cultivated and uncultivated organisms have comparable signal transduction capabilities. However, the percentage of one-component proteins in MAGs was found to be almost 20% fewer than in genomes, which indicates that individual MAGs can be different in this regard. Indeed, a MAG of a Margulisbacteria species (accession GCA_001771585.1), which represents one of the deepest branches of bacteria, contains 19 one-component systems, including three serine/threonine kinases that are relatively infrequent in bacteria, and >50 proteins that comprise two-component signal transduction. Thus, in-depth exploration of signal transduction in uncultivated microorganisms enabled by the new MiST release might challenge our current view on this important aspect of microbiology.

## Conclusions and future directions

MiST 4.0 provides signal transduction information of cultivated and uncultivated organisms within a single online resource, thereby laying the foundation for research in metagenomic signal transduction and comparative studies. A preliminary coarse-grained analysis of the protein sequences of the genomic and metagenomic parts of the database demonstrated a general similarity of the patterns of signal transduction systems in genomes and MAGs. However, many differences of various degrees have been uncovered between genomes and MAGs that showed that uncultivated organisms may have certain fundamental differences in signal transduction pathways compared to their cultivated counterparts. The differences revealed are just the tip of the iceberg, and the study of these unique features, requiring careful and systematic analysis, may lead to a new, deeper understanding of the phenomenon of signal transduction.

Future developments of MiST will include expansion of the metagenome section and enabling more robust functional annotation. The latter will involve developing new profile models for families with well-defined functions using conserved motifs for enzymatic activity and ligand binding (29,30). In a long term, we would like to transform MiST into an expertly curated database.

## Data Availability

The MiST 4.0 database is freely available for noncommercial use at https://mistdb.com. Users are not required to register or log in to access any of the features available in the database. GitHub repositories can be found at https://github.com/bioliners/projects (https://doi.org/10.5281/zenodo.8364134) and https://github.com/ToshkaDev/mist-web (https://doi.org/10.5281/zenodo.8364137). Docker images are available on Docker Hub at the following link: https://hub.docker.com/repositories/bioliners.
